# Lynch Syndrome in the Asian American Population: A Review of the Literature

**DOI:** 10.3390/cancers18091456

**Published:** 2026-05-01

**Authors:** Wai Park, Ashley Mochizuki, Lauren Gima, Joanne M. Jeter

**Affiliations:** Division of Clinical Cancer Genomics, Department of Medical Oncology, City of Hope Comprehensive Cancer Center, Duarte, CA 91010, USA; wpark@coh.org (W.P.); amochizuki@coh.org (A.M.); lgima@coh.org (L.G.)

**Keywords:** Asian Americans, Lynch syndrome, health disparities, colorectal cancer, endometrial cancer, gastric cancer, genetic testing

## Abstract

This paper aims to describe what is currently known about Lynch syndrome within Asian American populations. According to data collected by the US Census, as of 2022, 24.7 million people of Asian descent live in the United States. Cancer is the leading cause of death within this population, and as a result, it is crucial to identify ways that cancer can be identified at earlier and more treatable stages. Colorectal cancer is the third most common cancer diagnosis within the Asian American population, with an incidence of 37.1 per 100,000 Asian American men and 26.5 per 100,000 Asian American women. Lynch syndrome, the most common hereditary cause of colorectal cancer, has been incompletely described in this diverse population. This review addresses the available literature on the prevalence of Lynch syndrome in Asian American and Asian populations and differences in the manifestations of this syndrome between and within these populations, as well as in comparison to the non-Hispanic white population. Based on these differences, variances in screening rates, outcomes, and management strategies with respect to Asian ethnicity are also explored. Potential barriers to optimal management of Lynch syndrome in Asian American populations, with particular consideration of primary language and degree of cultural assimilation, are assessed. Future directions for research and recommendations to help address disparities or differences to optimize care for this group are also described.

## Simple Summary

This paper summarizes the current knowledge of the prevalence of and mortality from Lynch syndrome and its component cancers (including colon, endometrial, and gastric cancers) in Asian and Asian American populations. It compares Lynch syndrome diagnoses and outcomes in Asian American and non-Hispanic white populations and identifies potential avenues to optimize care. National guidelines for screening for these cancers in a Lynch syndrome population are reviewed, as well as perspectives on universal screening for colorectal cancers with immunohistochemistry and/or microsatellite instability testing. In addition, given the diversity within the Asian American ethnic group, it describes potential differences between and unique barriers for Asian American subpopulations. Lastly, it discusses opportunities to re-evaluate guidelines based on ethnic background and to increase research participation for this disease for Asian Americans collectively and for the varied subgroups that make up this population.

## 1. Introduction

Lynch syndrome (LS), also known as hereditary nonpolyposis colorectal cancer syndrome (HNPCC), is an autosomal dominant condition caused by germline heterozygous pathogenic/likely pathogenic variants (PVs/LPVs) in one of the DNA mismatch repair (MMR) genes, *MLH1*, *MSH2*, *MSH2*, and *PMS2*, or deletion in the 3′ end of the epithelial cell adhesion molecule (*EPCAM*) gene, leading to a loss of *MSH2* expression. Individuals with LS are at a significantly increased risk for colorectal, endometrial, and ovarian cancers at an earlier age compared to sporadic cancer cases. Other cancers, such as stomach, small bowel, urinary tract, biliary tract, brain (usually glioblastoma), skin (sebaceous adenomas, sebaceous carcinomas, and keratoacanthomas), pancreas, and prostate, are also more frequently reported in LS families.

To date, the understanding of the characteristics of LS-associated cancers is primarily based on studies of European/Caucasian populations. Accordingly, clinical practice guidelines are established based on the understanding of risks primarily in these populations. Cancer penetrance estimates in LS vary by the MMR gene as well as specific PVs in the same gene. As such, variability in MMR gene distribution may contribute to heterogeneity in risk assessment across different populations across the world. Moreover, penetrance among carriers varies substantially across ethnic, racial, and geographical factors. Given the substantial global variation in colorectal cancer risk in the general population, risk among individuals with LS may also differ by region. Understanding the similarities and differences in prevalence and phenotypic spectrum of MMR gene variants across different populations is crucial, as it can serve as a basis for population-specific surveillance and interventions with improved clinical outcomes. In this review, the available published literature on the incidence and characteristics of LS in Asian Americans, as well as available data in Asian countries, is summarized as the primary objective. Secondary objectives include the identification of disparities in practice and areas in which additional data are needed.

In this review, in alignment with the US Census Bureau definition, the term “Asian” refers to a person having origins in any of the original peoples of Central or East Asia, Southeast Asia, and the Indian subcontinent.

## 2. Population Prevalence of Lynch Syndrome in the United States

Estimates of Lynch syndrome prevalence in the American population and subpopulations vary significantly, and, in the case of data on Asian Americans, suffer due to small numbers. Epidemiologic data from the Colon Cancer Family Registry in the US, Canada, and Australia have estimated the prevalence of LS to be as high as 1 in 279 [1]. However, only 6% of the population in the study were self-reported Asians, and the prevalence in this population was not reported for this study. Similarly, the All of Us (AOU) Research Initiative study, which examined LS prevalence in a diverse US population, reported that approximately 1 in 354 individuals in the general US population have LS. While the population-based study included participants from various regions and backgrounds across the US, the carrier rate of LS in the Asian American population was not reported due to a participant count lower than 20 [2]. Three percent of the participants in the AOU study identified as Asian. More recently, the New York-based BioMe Biobank study estimated the overall prevalence of LS as 1:303 in self-reported South Asians and 1:757 in self-reported East Asian/Southeast Asians [3].

Larger international studies have approximated the prevalence rate based on worldwide data. The Genome Aggregation Database (gnomAD) v.2.1.1, an international database of exome and genome sequences, reported the estimated population prevalence of pathogenic variants in mismatch repair genes by ethnicity [4]. For East Asians (n = 9977), the prevalence of variants was 1:127, with 1:555 having null variants and 1:165 having missense variants. Of these, *MSH6* variants were most common, with fewer numbers of *MSH2* or *PMS2* variants and very few *MLH1* variants. In contrast, the South Asian cohort in the database (n = 15,308) had an estimated prevalence of 1:88, with null variants in 1:284 and missense variants in 1:127. For this cohort, *MSH6* variants were the most prevalent, followed closely by *PMS2* variants. Although these remain estimates based on limited data, these frequencies remain significantly higher than those reported in the American population, as cited above.

The reasons for these variations are not fully understood but likely have components attributable to a number of factors. First, individuals of Asian descent may be underrepresented in studies due to concerns about study participation, cultural issues regarding discussion of cancer or cancer screening, language barriers for consenting, or increased use of non-Western medical treatment. The considerable heterogeneity within the Asian American label must be considered as well, along with varying degrees of assimilation, differences in diet and other behaviors, and inequities in access to healthcare. As such, the true prevalence of LS among the Asian American population may not be accurately reflected in the reported numbers, or even by any single number.

## 3. Lynch Syndrome-Associated Cancers

### 3.1. Colorectal Cancer (CRC)

Among the spectrum of LS-associated malignancies, CRC accounts for the highest proportion of cancers. It is also the third most common cancer diagnosis within the general Asian American population, with an incidence of 35.3 per 100,000 Asian American males and 25.6 per 100,000 Asian American females from 2018 to 2021 [5].

Incidence rates of colorectal cancer in individual Asian countries are not uniformly reported by international agencies, likely due at least in part to the fact that the infrastructure needed for diagnosis varies significantly amongst these countries and that attention to colorectal cancer is eclipsed by competing cancer and non-cancer health concerns in some. In 2020, the World Health Organization (WHO) reported colorectal cancer incidence for the regions designated as Southeast Asia and West Pacific. For the Southeast Asia region, including 10 member states (Bangladesh, Bhutan, Democratic People’s Republic of Korea, India, Maldives, Myanmar, Nepal, Sri Lanka, Thailand, and Timor-Leste), colorectal cancer accounted for 6.2% of cancer incidence and 6.1% of cancer mortality in 2019 [6]. It was the fifth most common cancer incident and was tied for fifth for cancer mortality. For the West Pacific Region, 7.4% of incident cancers were CRC, and 5.7% of cancer deaths were due to this cause, making them the fifth most common incident cancer and the fifth in cancer mortality as well. However, this region consists of 38 member states, with some, such as Australia and New Zealand, that are not traditionally considered Asian countries. In 2022, country-specific data was reported by GLOBOCAN (see Table 1).

For specific countries, colorectal cancer mortality rates are available from GLOBOCAN from 2022 (see Table 2) [6]. Although these data can provide some insight into incidence in these countries, they may not correlate precisely, as incidence tended to be greater in higher-income countries that also had more resources for cancer treatment. In China, Japan, and the Republic of Korea, colorectal cancer was one of the 10 most frequent causes of overall deaths and one of the five most frequent causes of cancer deaths. Japanese women, in fact, had colorectal cancer as their most frequent cause of cancer deaths. In addition, colon cancer was the 10th most frequent cause of death in Tongan men. For men, mortality rates per 100,000 individuals were higher in many Asian countries (Democratic Republic of Korea, Guam, Independent State of Samoa, Japan, People’s Republic of China, Philippines, and Republic of Korea) than in the US. Mortality rates from CRC in women were higher than in their American counterparts in Guam, Japan, Samoa, Laos, and the Philippines as well. Other areas not specifically addressed in the SEER reporting categories, such as Singapore, also reported colorectal cancer as a frequent cause of overall mortality.

Data derived from the Centers for Disease Control and Surveillance, Epidemiology, and End Results Program (SEER) analyses of Asian American subpopulations showed substantial heterogeneity in CRC incidence across Asian American subgroups, with notably elevated incidence in Japanese Americans (see Table 3) [8,9,10]. When looking at mortality data, although the rates are lower amongst Asian American and Pacific Islanders than NHW populations, in general, the rate is higher than that for many Asian and Pacific Islander countries [11]. Of note, although mortality rates had decreased in NHW and in most Asian American subgroups from 2005 to 2020, they increased during this period for those of Korean and Vietnamese descent [12].

Characteristics of colon cancers in Asian populations have some significant differences compared to colon cancers in the US NHW population. Microsatellite instability is present in 12–16% of CRC in Western countries but only 6–7% of CRC in Japan [13,14,15,16,17].

LS is thought to account for 2.4–3.7% of CRC in Western countries. A single institution retrospective study reported a higher prevalence of LS (4.19%) among Asian participants diagnosed with CRC. However, the interpretation of these findings is limited by the relatively small sample size (n = 143) [18]. Japanese studies find LS prevalence in only 0.7–1.01% of CRC [19,20,21,22]. In China, one large study of universal screening of CRC showed that 8.7% of tumors had deficient mismatch repair and only 2.7% were ultimately diagnosed as being due to LS [23]. Among early-onset colorectal cancer (EOCRC) cases, the prevalence of LS is much higher: 6.7% of 3980 individuals with EOCRC in the US were found to have LS. The prevalence of LS among Asian participants in the cohort was comparable to that of NHW participants, 7.3% and 6.3%, respectively [24].

Currently, NCCN Clinical Practice Guidelines in Oncology (NCCN Guidelines^®^) serve as the primary framework for LS-related care in the US across diverse ethnic populations [25]. In comparison, one can look to available LS-related guidelines from Asian countries to identify differences in risk stratification, surveillance, and management that may have implications for optimizing care among Asian American populations with LS (see Table 4).

NCCN, as well as other major organizations, including the American College of Gastroenterology and the US Multi-Society Task Force on Colorectal Cancer, recommend universal screening of all CRC tumors with immunohistochemistry (IHC) for MMR and/or microsatellite instability (MSI) [25,30,31]. Previous studies have shown that clinical criteria, such as the Amsterdam Criteria and Bethesda Guidelines, often fail to identify patients with LS [32,33]. In addition, universal testing in all newly diagnosed patients with CRC has been shown to be cost-effective from the perspective of the US healthcare system, especially when IHC is used as a preliminary screening approach [34]. Likewise, the opinion of patients regarding universal screening has been shown to be mostly favorable, with positive impacts of screening for themselves and their families.

The Japanese Society for Cancer of the Colon and Rectum (JSCCR) updated its guidelines in 2024 to reflect the advances in genomic testing and the clinical practice setting in Japan [26]. The use of Amsterdam II (A2) criteria was emphasized in selecting patients for evaluation for LS. Although universal screening with microsatellite instability (MSI) or immunohistochemistry (IHC) for MMR proteins was only weakly recommended prior to this guideline update, the JSCCR does now advocate for this screening in all colon cancers, or all colon cancers diagnosed before the age of 70. Recommendations for regular colonoscopy starting at an early age in LS were similar to NCCN Guidelines^®^; the only difference was recommending colonoscopy every 1–2 years for patients with MSH6 PVs (NCCN V.1.2025 recommends 1–3 years for this group) [25]. In addition, they acknowledged but did not recommend the use of aspirin as chemoprevention for colon polyps. Previous guidelines from this group cited concerns about finding the appropriate dose and duration of aspirin treatment as well as balancing the risk of gastrointestinal bleeding [27]. For those with CRC, individualized decision-making was recommended for consideration of extended surgery. Immune checkpoint inhibitors were strongly recommended for recurrent or advanced CRC in Lynch patients.

In China, a clinical practice collaborative guideline was published by the Digestive Endoscopy and Colorectal Surgery Groups of the Chinese Medical Association, the Chinese Association of Gastroenterologists & Hepatologists, and the National Clinical Research Center for Digestive Disease in 2024 [28]. These guidelines make a weak recommendation for total or subtotal colectomy for patients with LS who have early-stage CRC; however, no other recommendations regarding the diagnosis or management of LS are made. CRC risk stratification is based on the Asia-Pacific Colorectal Screening questionnaire (APCS), which includes age, sex, history of first-degree relative with CRC, and smoking history [35]. Colonoscopy is recommended for higher-risk individuals; however, the participation rate for colonoscopy is noted to be low. A recent review of the status of LS care in China notes a lack of systematic registration and screening for this disorder, particularly outside of a few specialized hospitals [36]. The NCCN Guidelines are often used for guidance for clinical decision-making by Chinese healthcare providers. Universal screening for CRC and endometrial cancers is gradually being adopted. Most hospitals have access to IHC for MMR proteins, but only a few can provide MSI testing. The use of these tests to determine potential benefit from immunotherapy is increasing. Access to genetic counseling and testing varies widely, but these services are becoming more common. However, some experts report a high rate of patients declining germline testing. Initiation age and intervals for colonoscopy for *MLH1* and *MSH2* carriers are similar to NCCN V.1.2025 guidelines; however, for *MSH6* and *PMS2* carriers, colonoscopy is recommended to start at age 25–30 and repeat every 1–2 years.

### 3.2. Endometrial Cancer (EC)

EC is the second most common malignancy associated with LS and represents the most frequent extracolonic cancer among affected individuals. Based on the SEER Cancer Statistics, the incidence of EC is 28.3/100,000 persons, with incidence differing by race/ethnicity [37]. NHW individuals had an incidence of 27.6/100,000, while Asian American individuals had an incidence of 23.8/100,000 [37]. Although lower than that of other American subpopulations, Asian Americans are noted to have a more than 3-fold increase in annual incidence rate compared to White populations from 2001 to 2017 [38]. Likely in part due to acculturation and westernization, the relative incidence of EC in US-born Asians versus first-generation immigrants is significantly different, with US-born women having a higher incidence of EC than their non-US-born counterparts [39,40]. EC-associated mortality in the US has also been on the rise since the early 2000s, with the US mortality in 2022 reported as 3.1/100,000 [7]. Asian Americans have been shown to have lower mortality rates than NHW women, particularly for early-stage disease [41]. Importantly, categorizing all Asian and Pacific Islander individuals into one group may provide mischaracterizations and a distorted picture of the reality for some subgroups. EC-related mortality and incidence vary across Asian countries (see Table 1 and Table 2). Pacific Islanders have been consistently shown to have high incidence and mortality across different EC stages and histologic subtypes amongst these populations [41]. Out of the country-stratified data from GLOBOCAN, females from the Philippines had a similar mortality rate as females in the US, and Samoa was the only Asian country with a higher incidence and mortality rate than the US (see Table 1 and Table 2).

Based on the current literature, approximately 2–3% of EC are attributable to LS [32,42]. The prevalence of LS among Asian American patients with EC has been shown to be higher than in other ancestral groups [43]. A single institution study out of Memorial Sloan Kettering assessed 1625 patients with EC for PVs/LPVs in ≥76 cancer predisposition genes. Amongst all patients with a PV/LPV, 39/1625 (2.4%) had a PV/LPV in an LS gene. The highest rate of LS PVs/LPVs was identified in Asians (4.8%). The overall number of individuals testing positive in these ancestral groups, however, is notably low, necessitating confirmation of this finding within a broader cohort of individuals before drawing any conclusions [43]. Notably, a more recent study of 35,310 EC patients from the publicly available Myriad Collaborative Research Registry shows a consistent pattern with these data in much greater numbers, demonstrating that out of 1093 individuals of Asian ancestry in their cohort, 141 (12.9%) carried an LS PV/LPV, primarily in *MSH2* and *MLH1* [44]. Taken together, these studies suggest Asian Americans may be more likely to have LS as a contributing factor to their history of EC; however, consistent demonstration of this within larger cohorts would be beneficial while controlling for other risk factors of EC.

Tumor MMR analysis is important not only for the treatment of EC but also to identify candidates for germline genetic testing. Among Asian countries, studies of MMR deficiency in EC have shown variable rates of mismatch repair deficiency (dMMR). One Chinese study found that 26/150 (17.3%) of EC cases were dMMR. Conversely, a group from Pakistan looked at 126 cases of EC and noted abnormal IHC in 44.4% of cases [45]. Other Asian studies have noted abnormal IHC in about 28% of all EC [46]. More studies are needed to address this wide range, with country- or region-specific estimates being potentially more useful, given the heterogeneity of Asian populations.

Previous studies have shown that MMR status can be an independent prognostic factor for overall survival in EC [47]. However, several studies from Asian countries have shown dMMR was not correlated with disease outcomes, recurrence-free, or overall survival [48,49,50]. Additional studies are needed to identify if this is a consistent pattern amongst Asian populations.

In 2023, a Pan-Asian adapted version of the European Society for Medical Oncology (ESMO) Clinical Practice Guidelines was published and included representation from oncological societies of ten different Asian countries. The Pan-Asian panel of experts was mostly in agreement (80% of guidelines) with the ESMO guidelines [51]. Although these adapted guidelines mention that IHC/MSI status can serve as a screening test for LS, there is no explicit recommendation in the guidelines for germline genetic testing or screening related to LS. A consensus statement from the Korean Society of Gynecologic Oncology addresses MMR deficiency in the context of treatment decision-making; however, they do not explicitly provide a recommendation for genetic testing/counseling or guidance surrounding care for LS patients [52]. The Japan Society of Gynecologic Oncology guidelines from 2023 recommend lower gastrointestinal endoscopic surveillance for all patients with endometrial cancer diagnosed with LS and suggest genetic counseling for blood relatives of patients with endometrial cancer diagnosed with LS [53]. In China, the Expert Committee of Obstetrics and Gynecology of the Chinese Research Hospital Association recommends that all patients who have MMR-deficient endometrial cancers have “Lynch syndrome-related germline gene mutation testing and provide genetic risk counselling, assessment and follow up” with informed consent. They also recommend preimplantation genetic testing to “prevent inheritance” and recommend that, if fertility-preserving treatment is to be performed, patients are fully informed of the risks for themselves, as well as their offspring [54]. The Thai Gynecologic Cancer Society (TGCS) guidelines from early 2025 recommend that individuals with PVs/LPVs who are at increased risk for EC, including LS, consider annual transvaginal ultrasound and endometrial biopsy starting at age 30–35, following counseling on the risks, benefits, and limitations [55].

Available data suggest an increased prevalence of LS among Asian American individuals with EC. As such, there is a need for rigorous implementation of universal tumor testing and germline genetic evaluation to mitigate underdiagnosis of LS, not only in Asian American populations but also in Asian countries. However, it must be recognized that the large-scale implementation of genetic evaluation may be impractical in other resource-limited countries.

### 3.3. Gastric Cancer (GC)

Although GC is not one of the most common LS-associated malignancies in Western populations, it represents an important clinical concern among Asian and Asian American populations, as it is one of the most frequent and deadly malignancies in Asian countries.

According to GLOBOCAN data from 2022, GC was ranked fifth in both global cancer incidence and mortality [56]. The incidence rate of GC is highest in Eastern Asia, approximately double that of other world regions [57,58]. Country-specific GC incidence and mortality rates are available from GLOBOCAN from 2022 (see Table 1 and Table 2). GC incidence rates were higher in almost all Asian countries corresponding to SEER subcategories (Japan, Republic of Korea, Democratic People’s Republic of Korea, China, Vietnam, Samoa, Lao People’s Democratic Republic, Guam, India, Philippines, Cambodia) compared to the US, except for Pakistan. GC mortality rates were higher in all Asian countries corresponding to SEER subcategories as compared to the US. Mortality rates available from the WHO from 2021 also demonstrate that GC was one of the 10 most frequent causes of overall deaths in Azerbaijan, Bhutan, China, Japan, Kyrgyzstan, Mongolia, and the Republic of Korea.

A recent analysis of SEER data assessed GC incidence from 2000 to 2021 in Asian Americans as compared to other ethnic subpopulations in the US [59]. Asian and Black Americans showed the highest rate ratio of 1.94 compared to NHW, who consistently had the lowest rates of GC incidence. From 2000 to 2006, Asian Americans had the highest incidence rates compared to all other subgroups. There are notable differences in GC incidence rates amongst Asian American subgroups. SEER data from 1990 to 2014 demonstrated that GC incidence was highest in Koreans, Japanese, and other Pacific Islanders (Samoan, Guamanian/Chamorro) as compared to NHW, though rates were still elevated in Chinese, Hawaiian, Filipino, Southeast Asians (Vietnamese, Laotian, Kampuchean/Cambodian), and Asian Indian/Pakistanis [60]. This trend was mirrored in the California Cancer Registry, where Korean Americans, particularly males over age 50, had the highest rates of GC (12–14-fold increased) compared to NHW [61].

SEER data on cancer-related mortality rates in the US from 2019 to 2023 showed that Asian/Pacific Islander males died from GC 49% more often than males in the general population, and Asian/Pacific Islander females died from GC 67% more often than females in the general population [62]. Mortality rates from cancer-related deaths in Californians from 2012 to 2017 reflect that almost all Asian American subgroups (Korean, Japanese, Chinese, Filipino, Native Hawaiians and Other Pacific Islanders, Vietnamese, Southeast Asians) had higher mortality from GC as compared to NHW (mortality rate ratios [MRR] varied from 1.05 to 4.4). The only Asian American subgroup that demonstrated lower mortality rates (MRR 0.48) was South Asians [63].

The “migrant effect” describes the trend of immigration and acculturation potentially shifting cancer incidence rates closer to that of the host population [64]. This effect may have been observed amongst Japanese immigrants in Hawaii as compared to Caucasians in Hawaii and Japanese in Japan, in which each successive generation residing in the US had a 33% reduction in risk for GC [64,65]. Data from the California Cancer Registry also shows that GC incidence rates are consistently higher (~2–3 fold) among foreign-born Korean, Chinese, and Japanese individuals as compared to their US-born counterparts [66].

Elevated GC incidence rates in Asian countries are likely due to factors such as higher rates of *H. pylori* infection, dietary patterns, and increased tobacco use [56,58]. In 2020, GC attributable to *H. pylori* infection accounted for 4.4% of all cancers globally, with the highest rates of these cancers in South-Central Asia and Eastern Asia [67]. Globally, cases from China and Japan made up 42% and 14% of H. *pylori*-attributable GC in 2020, respectively [67]. As such, *H. pylori* irradiation has been covered through insurance since 2015 in Japan [68]. The Republic of Korea’s national insurance also covers *H. pylori* treatment for certain indications, such as gastric ulcer, MALT lymphoma, post-treatment of early-stage GC, ITP, and post-resection of gastric adenomas [69].

Data are limited regarding LS-associated GC in Asian countries and primarily stem from research in the Japanese population. In a JCCR cohort of 69 patients with LS and CRC, 15.9% were also found to have GC [70]. Many of these LS patients (43/69, 62%) also reported a family history of GC in a first- or second-degree relative. GC was a major cause of death in first-degree relatives of Japanese LS patients, after CRC and EC [71].

Japanese LS patients may also be at higher risk of multiple GCs. Among a different cohort of 96 Japanese LS patients at a single institution, 32 GCs were detected in 15 patients [72]. The cumulative risk of Japanese individuals with LS developing GC at 70 years was 31.3%. Most of these GCs (82%) demonstrated microsatellite instability. This observed GC risk exceeds the currently reported lifetime estimates of up to 9%, largely based on European and North American LS cohorts.

The high incidence of GC in Japanese LS patients is a crucial factor in how patients are potentially screened for LS diagnosis. When modifying A2 criteria to include GC, 111/4056 patients enrolled in a Japanese CRC registry met these modified A2 criteria, and 64 were identified to have LS [73]. A total of 17% (11/64) of these LS patients would have been missed if GC were not included in the modified A2 criteria.

While the LS-associated GC risk thus far described in the Japanese population is valuable in understanding potential differences in LS presentation amongst Asian populations as compared to North American and European populations, this information cannot be generalized to other Asian countries, and it is unknown whether similar trends could be observed in other Asian populations. Furthermore, the Japanese LS GC cohorts described above were primarily describing relatively small cohorts from single institutions, and larger population-based studies are needed to determine the potential generalizability of this data. Studies of larger populations from Asian countries and US-based studies that include more Asian Americans will be crucial to determine if population-specific GC screening for LS patients is warranted.

Several guidelines currently support the use of upper endoscopy (EGD) for surveillance for GC, but recommendations vary concerning risk identification, the age at which surveillance should begin, and the frequency of repeat EGD (see Table 5).

### 3.4. Other LS Cancers

Owing to the relatively low frequency of other cancers in LS, as well as the underrepresentation of Asian Americans in hereditary cancer research, there are limited data available for other LS-related cancers in Asian American populations.

#### 3.4.1. Upper-Tract Urothelial Carcinoma (UTUC)

UTUC represents the third most frequent malignancy, following CRC and EC, in LS in Western cohorts. In general, the lifetime risk of UTUC in Lynch syndrome patients is reported as high as 28%, with the highest risks in those with *MSH2* pathogenic variants [25]. Risks for individuals with *MLH1* or *MSH6* are up to 5.5%, and there does not appear to be a significantly increased risk of UTUC with *PMS2* pathogenic variants [25]. As with CRC and EC, LS-associated UTUCs are generally diagnosed 10–15 years younger and have less exposure to tobacco compared to patients with sporadic UTUC [78]. The identification of LS can have implications for the management of UTUC patients, including consideration of nephron-sparing surgical approaches and improved responses to immunotherapy.

No published data on LS-associated UTUC specifically in Asian American populations have been found to date. A multicenter cohort study from Japan recently reported that the cumulative incidence of UTUC at 70 years of age was 10–28% among LS patients, with the highest risk associated with *MSH2* PVs [79]. The data is consistent with findings from the Prospective Lynch Syndrome Database, in which the cumulative incidence for UTUC by age 75 ranges from 8% to 25%, with the highest risk in those with *MSH2* PVs [80].

A recent retrospective study from an American commercial laboratory shows the prevalence of LS-associated germline alterations in UTUC patients is approximately 1.8%; ancestry-specific data was not reported [81]. Similar prevalence rates of LS have been reported in UTUC cases in Japan and China [82,83].

The high sensitivity of MMR protein loss in UTUC cases associated with LS has been previously suggested. A single-center retrospective study in China noted that all patients in the cohort with LS and UTUC had MSI-high tumors or loss of MMR-corresponding protein on IHC [84]. It should be noted that out of 309 patients who were included in this study, 12 were identified to have Lynch syndrome, and *MSH2* was the most likely MMR gene in which a pathogenic variant was identified (10/12). Both the American Urological Association and European Association of Urology recommend MMR testing for patients with early-onset UTUC or a relevant family history [85,86]. Universal tumor testing in UTUC may be helpful in identifying Asian patients with LS, as there are many possible common confounders, such as a higher prevalence of smoking history and aristolochic acid (an ingredient found mainly in herbal remedies and traditional Chinese medicine) exposure in this population.

#### 3.4.2. Ovarian Cancer (OC)

OC represents a clinically significant extracolonic malignancy in LS. Recently, a US-based commercial laboratory collaborative research registry study evaluated ancestry-based differences in the germline landscape of >80,000 patients with epithelial OC [87]. The study found the highest prevalence of LS (1.3%) among self-reported Asians, followed by self-reported Whites (1.0%). Notably, among all Asian patients with LS (n = 37), *PMS2* PVs were significantly higher in Asians (n = 12) compared to other ancestry groups. Similar findings were reported in an unselected cohort of 230 Japanese patients with OC, in which *PMS2* PVs were more frequently observed compared to *MLH1* and *MSH2* [88]. These findings may be attributable to several factors. First, LS-associated OCs are more likely to be non-serous histologic subtypes, such as endometrioid and clear cell carcinomas, in both European and Asian (Japanese/Hong Kongese) populations [89,90,91]. Previous studies have reported that endometrioid and clear cell carcinomas are more common in Asians, irrespective of birthplace (American-born versus foreign-born), compared to their Caucasian counterparts, hence potentially introducing an ascertainment bias [92]. Second, *PMS2* is the most frequently identified PV among LS-associated genes, which may further influence the observed distribution. Nonetheless, further investigation of *PMS2*-associated OC risk in large Asian American cohorts is warranted to inform population-specific, evidence-based risk management strategies in this population. Current data, mainly derived from European populations, indicate that the risk for OC in women with *PMS2*-associated LS is not much higher than that of the general population. As such, NCCN V.1.2025 guidelines no longer include OC-specific management recommendations in *PMS2* carriers [25].

#### 3.4.3. Biliary Tract Cancer (BTC)

Gallbladder cancer and cholangiocarcinoma, collectively known as BTC, are other extracolonic LS-associated cancers with high mortality rates. Although LS-associated BTC is reported to have higher overall survival rates compared to sporadic cases, 5-year survival remains low at 29% [80]. In the US, non-Hispanic Asian/Pacific Islander groups have higher intrahepatic bile duct cancer incidence and mortality rates than NHW [93]. Cumulative risk of BTC among LS carriers (*MLH1* in particular) has been reported as high as 7.2% by age 70 in a Japanese study as compared to current risk estimates of 3.7% in studies with primarily Caucasian participants [94]. Rates of cholangiocarcinoma and biliary tract cancers are estimated to be even higher in areas of Southeast Asia, such as Thailand and Cambodia, due to endemic liver flukes in these countries, although accurate data are difficult to obtain for this malignancy due to diagnostic difficulties and frequent combination with hepatocellular carcinoma for summary statistics. This level of risk is similar to that for cholangiocarcinoma in individuals with primary sclerosing cholangitis (5–20%), for which both the American Gastroenterological Association and American Association for the Study of Liver Diseases 2023 practice guidelines recommend routine surveillance with MRI/MRCP [95,96]. There is currently no established surveillance protocol for BTC in LS patients. Further studies are needed to confirm if BTC risk is elevated among Asians and Asian Americans with LS to better inform surveillance strategies.

#### 3.4.4. Brain Tumors

Overall, brain tumors are rare in LS. According to the Prospective LS Database, cumulative brain tumor incidence was 0–7.7% by age 75 years, depending on the affected MMR gene and sex. The highest risk has been found in individuals with *MSH2* PVs/LPVs [97].

As for the prevalence of LS among brain tumors, Korean researchers reported four cases with LS among a series of 740 brain tumors (0.5% of the total) [98]. Although limited by a small sample size, this result is comparable to previously reported prevalence of LS among brain tumors in France and the US [99,100]. The most common LS-associated brain tumor across these studies was glioblastoma (GBM).

Due to the small number of cases and short follow-up duration, no statistical difference in progression-free survival (PFS) was seen between MMR-deficient gliomas and MMR-proficient gliomas in a Korean study. However, an overall trend for decreased PFS was noted in MMR-deficient gliomas [98]. Similar findings have been reported with shorter median survival in LS-associated GBM compared to an age-adjusted sporadic GBM cohort in studies from the US with predominantly Caucasian participants [101,102].

## 4. Addressing Healthcare Disparities Among Asian American Populations with Lynch Syndrome

Despite being one of the fastest-growing and most heterogeneous racial and ethnic groups in the US, Asian Americans experience persistent disparities in cancer care that are not obvious. The substantial heterogeneity among Asian Americans—including sociocultural characteristics, immigration patterns, and socioeconomic status—obscures critical disparities in cancer risk assessment, screening, management, and health outcomes across this population.

Disparities can start at the point of referral for genetic testing. A large observational study by Kurian et al. noted that among patients diagnosed with cancer, Asian patients had lower genetic testing rates than NHW patients [103]. When controlling for age, cancer type, and year, the genetic testing rate amongst Asian patients was 6% compared to 8% amongst White patients. This difference was exacerbated for certain cancer types, with testing probability among patients with male breast cancer, female breast cancer, or ovarian cancer occurring for 22% of Asian patients and 31% of White patients [103]. The authors acknowledge that there are many possible explanations for this finding, including individual preferences and insurance coverage.

Additionally, the lower rate of genetic testing may in part be due to low provider referral rates for Asian American patients [104]. A meta-analysis of ovarian cancer patients reported that 23% of Asian patients were referred to genetic counseling compared to 40% of White patients [105]. In addition to race/ethnicity affecting referral rates, English language proficiency has been shown to be significantly associated with referral [104]. Another factor contributing to the lower genetic testing rate among Asian Americans may be patients’ lack of awareness of genetic testing and potential benefits. This knowledge deficit could be attributed to language/communication barriers, as well as cultural differences that may decrease testing uptake [106]. While a substantial proportion of the general public lacks awareness of genetic testing, the National Cancer Institute’s 2017 Health Information National Trends Survey estimated that Asians were even less likely to be aware of genetic testing (OR 0.31) compared to NHW respondents [107]. These discrepancies demonstrate the strong need for education programs that are linguistically and culturally customized for various subgroups of the Asian American population to increase awareness of genetic testing.

Performing genetic cancer risk assessment is another potential challenge in Asian American populations. Obtaining an accurate and comprehensive family history is crucial, as many existing guidelines for germline genetic testing rely heavily on family history. The A2 criteria are largely dependent on a multigenerational family history of cancer; however, these criteria have a sensitivity rate of only 22–45% for the identification of LS. Considering that roughly half of Asian Americans are foreign-born, several challenges exist that may obscure patterns of cancer in the family. Relatives from earlier generations are from countries with different healthcare systems, limited documentation, or inconsistent medical record-keeping practices; as such, family health histories are often incomplete or difficult to verify, hindering accurate risk assessment. Moreover, variations in cancer screening practices in Asian countries may lead to underdiagnosis, further reducing the sensitivity of the A2 criteria.

Importantly, cultural norms and beliefs may hinder willingness to disclose and discuss family history among certain Asian subgroups. In one study, many Chinese participants explained that asking about family members’ disease history can be considered impolite, and discussing disease among families is perceived as taboo [108]. Moreover, the former national “one-child policy,” which led to small family sizes in China, also presents an additional challenge by potentially truncating family structure for hereditary cancer risk assessment among Chinese American families.

Screening practices in Asian Americans also demonstrate some disparities. In 2020, only 55% of Asian Americans over age 50 were found to be up to date on colon cancer screening as compared to 66% of the NHW population [109]. In addition, Asian Americans were more likely to perform stool testing and less likely to have a colonoscopy than the general population. Analysis of immigration status also showed that Asians who immigrated to the US were less likely to be up to date on colon cancer screening than Asians born in the US.

These factors provide additional considerations that may need to be included in assessments by healthcare providers, guideline determination by subspecialty associations, and evaluation of testing coverage criteria by insurers. Given these unique challenges in assessing hereditary cancer risk among Asian American populations, as well as the clinical evidence that individuals of Asian ancestry may be over-represented in some diseases associated with Lynch syndrome, current germline testing criteria require adaptation to enhance the identification of high-risk individuals.

Furthermore, disparities may stem from the lack of diverse racial and ethnic control populations in genomic datasets. The report of variant of uncertain significance (VUS) rates varies, ranging from 10 to 40% in patients undergoing genetic testing for hereditary disorders [110,111,112]. Most large reference databases used for variant interpretation are disproportionately derived from individuals of European ancestry. Underrepresentation of minority ethnic groups, including Asians, in genetic research and limited availability of ancestry-specific segregation data can cause problems in determining the pathogenicity (or lack thereof) for variants that may be specific or more common in those populations. As such, rates of VUS findings are higher in Asian patients compared to White patients tested for hereditary breast and ovarian cancer and Lynch syndrome in a US-based population (OR: 2.44; CI: 1.18–5.15) [113]. Ndugga-Kabuye et al. evaluated VUS prevalence in 50,000 patients from a single commercial laboratory. A higher prevalence of VUS in *BRCA1*/*BRCA2* and LS genes was noted in Asians and Pacific Islanders compared to those of European ancestry [114]. Although the American College of Medical Genetics emphasizes that VUS should not be used in clinical decision-making, some clinicians may overinterpret these findings, resulting in unnecessary surveillance or inappropriate risk-reducing interventions, while others may discount the result entirely without adequate counseling or follow-up. Moreover, VUS may require ongoing communication between patients and providers to review interpretation updates over time. The onus of this communication is an additional responsibility, potentially leaving patients vulnerable to being lost to follow-up with the VUS unresolved. The disproportionate rate of VUS in Asian American populations also adds to disparities in patient burden. Population-specific reference genome datasets in Asian populations are essential for characterizing genetic variation and classification in this understudied group.

Due to the limited availability of subgroup-specific data, many studies included in this review reported aggregate data on Asian American populations. This is an acknowledged limitation, which prevents the full discussion of the meaningful heterogeneity that exists among Asian American subgroups. In addition to genetic differences, variable cultural practices, environmental exposures, socioeconomic status, and health behaviors may have important implications on cancer risk and survival outcomes among different ethnic subgroups. This heterogeneity has already been demonstrated in cancer screening, incidence, and mortality differences of CRC, EC, and GC among different Asian American subpopulations by several groups [8,12,115,116,117,118].

Moreover, the conduct of this review is by its nature limited in that, for practical reasons, it can only include English language publications. As such, data reported in languages other than English may have inadvertently been omitted.

Considering the population differences and lack of specific data in these populations, there is a critical need for future studies of LS in Asian Americans. Intentional recruitment of Asian American populations using disaggregated data will help to better characterize subgroup-specific genetic variant distributions and improve risk stratification, treatment, and clinical outcomes. Identifying and addressing the barriers to participation in research among this group is vital. Liu et al. cited that lack of trust in health research may contribute to lower recruitment among Asian Americans compared to other racial/ethnic groups, even after adjustment for age, sex, education level, and health conditions [119]. In addition to having a diverse research team, choosing a venue where Asian Americans congregate, such as faith-based organizations, Asian community centers, etc., could help improve engagement and trust within the Asian American community. The same study also noted that a lower percentage of Asian Americans preferred English as the language to receive health information. As such, use of culturally and linguistically appropriate recruitment materials may help mitigate language-related barriers to participation.

## 5. Conclusions

This literature review reveals a substantial paucity of data characterizing LS among Asian American populations, limiting the generalizability of current risk estimates and clinical recommendations to this understudied group. Available evidence suggests that Asian Americans with LS may have differences in certain disease characteristics, including a disproportionately elevated risk of GC, considering the higher baseline incidence in Asian countries. In this context, existing surveillance and management guidelines in the US may warrant reconsideration to account for this compounded risk among Asian Americans with LS. Specifically, consideration may be given to:

1. Assessment of existing recommendations for upper endoscopy screening in LS to determine appropriateness for Asian American populations in terms of starting age and frequency of this procedure;
2. Evaluation of the efficacy and safety of aspirin as polyp chemoprevention in East Asian and South Asian populations;
3. Consideration of dietary interventions to reduce gastric cancer risks in Asian and Asian American populations;
4. Assessment of the utility of measures other than colonoscopy (e.g., stool tests) as a modality potentially more acceptable and more accessible to populations of Asian descent.

On a more systemic level, future investigations should prioritize inclusion of Asian American individuals with careful attention to the heterogeneity across ethnic subgroups. Specifically, the following measures may be beneficial:

1. Culturally tailored information and education in Asian languages;
2. Use of community health promoters or navigators to improve knowledge about cancer risk assessment and prevention outside of the healthcare setting;
3. Recognition, recruitment, and analysis of the different subgroups under the umbrella of the Asian American population in epidemiologic and clinical studies;
4. Active inclusion of health disparity and diversity experts in guidelines, outreach efforts, and prevention studies;
5. International data sharing initiatives, such as integrating datasets from Asian countries, to significantly expand diversity within the evidence base for variant interpretation.

With an improved understanding of differences in incidence and cultural factors amongst Asians and Asian Americans, management of LS can be further tailored to the unique needs of these populations.

## Figures and Tables

**Table 1:** Cancer incidence from GLOBOCAN 2022 (version 1.1) data with countries corresponding to SEER Asian American categories [7].

Country	CRC Male	CRC Female	EC	GC Male	GC Female
Cambodia/Kampuchea	14.2	11.5	3.1	5.4	3.2
Democratic People’s Republic of Korea	23.8	15.5	4.5	22.1	8.1
Guam	18.4	13.8	14.9	7.1	5.2
Independent State of Samoa *	33.9	17.7	26.2	14.1	9.2
India	6.1	3.7	2.5	6.1	3.0
Japan	45.5	28.5	14.8	40.9	15.9
Lao People’s Democratic Republic	15.9	12.5	3.3	14.9	8.1
Pakistan	6.3	4.5	3.8	4.0	3.0
People’s Republic of China **	24.7	15.7	6.8	19.5	8.3
Philippines	25.6	17.0	9.9	5.3	3.3
Republic of Korea	33.8	20.1	7.6	38.4	16.9
Vietnam	17.5	11.0	7.8	18.6	9.2
United States ***	30.1	24.2	22.5	5.1	3.2

Values per 100,000. * Data not reported separately for American Samoa. ** Data not reported separately for Taiwan. *** Data not reported separately for Native Hawaiians.

**Table 2:** Cancer mortality from GLOBOCAN 2022 (version 1.1) data with countries corresponding to SEER Asian American categories [7].

Country	CRC Male	CRC Female	EC	GC Male	GC Female
Cambodia/Kampuchea	8.9	7.1	1.1	4.6	2.7
Democratic People’s Republic of Korea	13.8	9.0	0.98	18.2	6.5
Guam	11.6	7.6	2.3	4.8	3.6
Independent State of Samoa *	16.5	8.1	9.5	11.2	9.2
India	3.6	2.2	0.96	5.5	2.7
Japan	14.2	8.8	1.9	10.8	4.4
Lao People’s Democratic Republic	9.6	7.3	1.1	12.8	6.9
Pakistan	3.6	2.5	1.3	3.5	2.6
People’s Republic of China **	10.9	6.5	1.1	13.8	5.3
Philippines	13.4	8.8	3.1	4.5	2.7
Republic of Korea	11.7	6.0	0.85	8.9	4.7
Vietnam	8.7	5.4	2.1	15.2	7.4
United States ***	9.5	6.4	3.1	2.1	1.2

Values per 100,000. * Data not reported separately for American Samoa. ** Data not reported separately for Taiwan. *** Data not reported separately for Native Hawaiians.

**Table 3:** Colorectal cancer (CRC) incidence and mortality in Asian American subgroups (SEER and CDC data).

	Subgroups	Rate per 100,000	Notes
Incidence (SEER 2004–2008) [8]	Asian Indians/Pakistanis (M/F)	23.4/18.8	Lowest incidence among Asian subgroups
	Japanese Americans (M/F)	66.6/43.0	Highest incidence; exceeds NHW
	NHW (M/F)	54.0/40.6	—
Incidence (SEER 2012–2016) [9,10]	Asian/Pacific Islanders (overall)	30.0	
	Japanese American (M/F)	62.2/39.4	
	NHW	38.6	
Mortality (CDC 2013–2017) [9]	Asian American/Pacific Islanders (M/F)	11.4/8.1	Higher when compared to Asian countries of origin (see Table 2)
	NHW (M/F)	16.3/11.7	

**Table 4:** Comparison of LS management recommendations for CRC.

	Universal Screening of Colorectal Cancers	Aspirin	Colonoscopy Start/Frequency			
			*MLH1*	*MSH2/EPCAM*	*MSH6*	*PMS2*
NCCN [25]	Yes	Consider on individual basis for *MLH1*, *MSH2*, *MSH6*, less so for *PMS2*	Start at 20–25; repeat every 1–2 years	Start at 20–25; repeat every 1–2 years	Start at 30–35; repeat every 1–3 years	Start at 30–35; repeat every 1–3 years
Japan [26,27]	Yes, or all colon cancers prior to age 70	Not recommended due to bleeding concerns, dosing issues	Start at 20–25; repeat every 1–2 years	Start at 20–25; repeat every 1–2 years	Start at 30–35; repeat every 1–2 years	Start at 30–35; repeat every 1–3 years
China [28]	Increasing but not widely available	N/A	Start at 20–25; repeat every 1–2 years	Start at 20–25; repeat every 1–2 years	Start at 25–30; repeat every 1–2 years	Start at 25–30; repeat every 1–2 years
Korea [29]	Conditional for all patients	N/A	N/A	N/A	N/A	N/A

**Table 5:** Comparison of GC screening recommendations by country.

Country	Population Screening Recommended?	Specific Recs for LS-Associated GC Screening?	Age to Initiate Screening	Screening Interval
US [25]	No *	Yes	30–40 years	2–4 years
Japan [74]	Yes	Yes	30–35 years for LS screening 50 years for population’ screening	Annual
Republic of Korea [75]	Yes	No	40 years	2 years
China [76]	Yes, in certain high-risk rural and urban areas	No	40–69 years	6 months–3 years

* The American Gastroenterological Association recommends screening via endoscopy for certain individuals at higher risk, including first-generation immigrants from high-incidence regions (East Asia, Eastern Europe, Latin America), those with a family history in a first-degree relative, those with H. pylori infection, and those with a hereditary predisposition or other high-risk lesions, such as incidentally identified intestinal metaplasia [77].

## Data Availability

No new data were created or analyzed in this study.
